# Effects of Using Pozzolan and Portland Cement in the Treatment of Dispersive Clay

**DOI:** 10.1155/2013/547615

**Published:** 2013-06-24

**Authors:** A. H. Vakili, M. R. Selamat, H. Moayedi

**Affiliations:** ^1^School of Civil Engineering, Universiti Sains Malaysia, 14300 Nibong Tebal, Penang, Malaysia; ^2^Department of Geotechnics and Transportation, Faculty of Civil Engineering, Universiti Teknologi Malaysia, 81300 Johor, Malaysia

## Abstract

Use of dispersive clay as construction material requires treatment such as by chemical addition. Treatments to dispersive clay using pozzolan and Portland cement, singly and simultaneously, were carried out in this study. When used alone, the optimum amount of pozzolan required to treat a fully dispersive clay sample was 5%, but the curing time to reduce dispersion potential, from 100% to 30% or less, was 3 month long. On the other hand, also when used alone, a 3% cement content was capable of reducing dispersion potential to almost zero percent in only 7 days; and a 2% cement content was capable of achieving similar result in 14 days. However, treatment by cement alone is costly and could jeopardize the long term performance. Thus, a combined 5% pozzolan and 1.5% cement content was found capable of reducing dispersion potential from 100% to zero percent in 14 days. The results indicate that although simultaneous treatment with pozzolan and cement would extend the required curing time in comparison to treatment by cement alone of a higher content, the task could still be carried out in a reasonable period of curing time while avoiding the drawbacks of using either pozzolan or cement alone.

## 1. Introduction

Dispersive clays have been found in various types of climates and in various locations in Australia, Brazil, Iran, New Zealand, The United States, Thailand, Mexico, Vietnam, South Africa, and many other countries [1–3]. Dispersion potential, measured in terms of percent dispersion, is a physicochemical phenomenon that is mostly influenced by minerals in the clays and chemical contents of pore water [4–6]. Whenever dispersive clay is exposed to water, especially with low salt concentration, the particles separate from each other, become dispersed, and get washed away such as in the progressive erosion phenomenon known as piping [7–12]. The origins of piping which could be cracks due to shrinkage, unequal settlement, or structural fractures need to be avoided at an early stage and the construction materials for earth dams, embankments, and foundations need to be erosion resistant [7]. 

Dispersive clays usually have high percentages of exchangeable sodium ion—Na^+^—which is susceptible to replacement by calcium and aluminum ions—Ca^2+^ and Al^3+^ [2, 9]. Changes in soil characteristics during chemical treatment are likely due to the cation exchange Na^+^ with Ca^2+^ and Al^3+^, reduction in the thickness of diffused double layer, and the subsequent reduction in the repulsive forces of the clay particles [3]. 

In order to recognize a dispersive clay or to measure dispersion potential, researchers have recommended physical and chemical tests such as double hydrometer test in determining percent dispersion (ASTM D 4221-99, 2000) [13], pinhole test in determining final flow rate through sample (ASTM D 4647-93, 2000) [14], and chemical tests in determining related indexes namely electrical conductivity (EC), sodium absorption ratio (SAR), and percent sodium (PS) (ASTM D 4542-95, 2000) [15]. Dispersion potential lessens with rising EC, while EC rises with increasing electrolyte or cation concentration in clay. Dispersion potential rises with increasing PS in clay [16–19]. 

The criteria for evaluating dispersion potential of a clay sample using results from the double hydrometer test have been presented by Sherard and Decker (1977) [20]. Clay with dispersion potential less than 30 percent can be considered as non-dispersive and thus erosion resistant. However, fully non-dispersive clay or one with zero percent dispersion potential is absolutely more preferred as construction material of certain projects.

The addition of chemical stabilizers to a dispersive clay would cause several chemical reactions including cation exchange reaction, flocculation-agglomeration reaction, pozzolanic reaction, and hydration reaction [21]. In the construction industry, the handlings of dispersive clays have often involved sole treatment additives such as lime, gypsum, or aluminum sulfate [2, 9, 22]. However, there were drawbacks in using any of these alone, for example, use of lime has caused volume increase in the treated soil due to carbonation reaction and sulfate attack, and thus decrease in strength. In preventing such problem, suitable curing times were allocated, but simultaneous uses of pozzolan, slag, and sulfate resistance cement together with lime have been regarded as solutions to prevent failures resulting from the use of lime alone [21, 23].

Pozzolan is a finely divided siliceous, or siliceous and aluminous, material which by itself possesses little or no cementing value. In the presence of moisture, however, it will chemically react with lime at ordinary temperatures to form cementing compounds. Natural pozzolans from different geographical regions nevertheless have diverse properties due to their respective chemical compositions which could lead to varying results in the products treated by these additive materials [3]. 

Portland cement has also been used extensively in treating clays in the construction industry. Because of the existence of lime in most cements, the latter would not be any better than the former if used alone as additive in stabilizing dispersive clays. For this reason and the fact that, when used alone, certain pozzolans also have not been effective in the treatment of dispersive clays, simultaneous use of pozzolan and cement of various mix ratios was introduced in this research. 

To measure dispersion potential and relevant properties of various test mixtures with respective curing periods, double hydrometer tests, pinhole tests, chemical tests, and unconfined compression tests were carried out in this study. The pinhole test was specifically appealing because of its exactness in modelling seepage conditions in actual engineering structures [12]. 

## 2. Materials and Methods

The clay sample, a Montmorillonite (Mt), classified as Low Plasticity Clay (CL) by the Unified Soil Classification System (USCS), was sourced from an extensive deposit of clay in Azerbaijan, Northwestern Iran. All incoming material passed Sieve number 10 (2.00 mm), while 98 percent of it passed Sieve number 200 (0.075 mm). In the natural state, the dispersion potential of the clay was generally more than 50 percent and thus dispersive, as it was widely known to be. 

In the laboratory the clay was altered to achieve a 100 percent dispersion potential. Different percentages of sodium hexametaphosphate—a dispersion agent—were added to various batches of the sample. The curing was carried out for 24 hours at optimum moisture content to allow equilibrium between salt contents in the pore water. The optimum moisture content in curing was as in a proctor test—water content at maximum dry density. 

**Figure 1:**
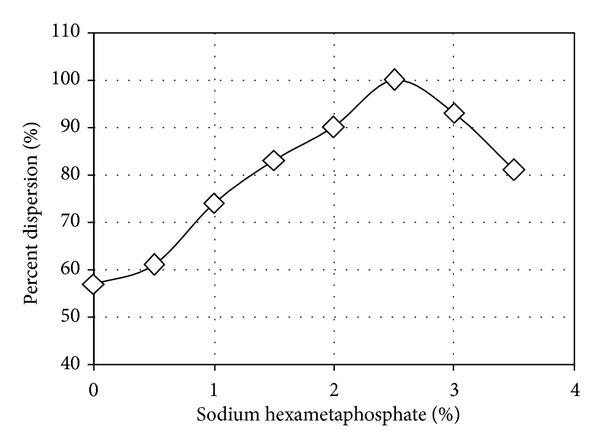
The effect of adding sodium hexametaphosphate on dispersion potential of clay sample from Azerbaijan, Iran.

The dispersion potential after curing was determined by double hydrometer tests which have produced results shown in Figure 1. One hundred percent dispersion was achieved with 2.5 percent sodium hexametaphosphate added to the sample, and the mix was thus considered fully dispersive and extremely erodible. The subsequent tests carried out on the sample have produced results shown in Table 1.

To this mix, the treatment commenced in the study by adding pozzolan and Portland cement, separately and simultaneously, and having the treated clays tested by double hydrometer test, pinhole test, and chemical test in order to assess any reduction in dispersion potential or improvement in resistance to erosion.

The pozzolan used in this research was the natural type used in Estahban cement factory, Estahban, South-Central Iran, which was sourced from Sirjan, a district about 180 Km to the East. In this case, the distance between dispersive clay deposit in Azerbaijan and pozzolan source in Sirjan was therefore more than 1200 km, but pozzolans were also supplied by others in the country although with varying properties. 

The pozzolan as provided by the supplier was in granular form and needed grinding in the Los Angeles abrasion apparatus before being put through the number 200 sieve. The specific gravity [27], Gs of pozzolan used was 2.60, which was lower than the Gs, of clay, at 2.79. The subsequent soil stabilization processes were carried out based on standard compaction procedure of ASTM D 698-91 (2000) [28].

For tests involving single additive, pozzolan amounting to 2, 4, 5, 6, and 8 percent of dry sample weights was used for the respective mixes. Curing times were 1, 7, 14, 35, and 90 days. Also, Portland cement amounting to 1, 2, 3, and 4 percent of dry sample weights was used for the corresponding mixes. Curing times were 1, 7, 14, and 35 days. For tests involving simultaneous pozzolan and Portland cement additives, the mixing percentages were 2, 5, and 8 for the pozzolan and 1.5 for the cement. Curing times were 1, 7, 14, and 35 days. 

The tests carried out were double hydrometer, pinhole, and chemical. The chemical tests have involved determining concentrations of major cations including calcium, magnesium, sodium, and potassium, in milliequivalents per liter unit of saturation extract. Thereafter, sodium absorption ratio (SAR), total dissolved salts (TDS), and percent sodium (PS) were calculated using related equations. Variation of electrical conductivity (EC) was also determined experimentally from the chemical tests. 

Finally, to further verify optimum pozzolan and cement contents in the treatment of dispersive clays, singly and simultaneously, unconfined compression tests in accordance with ASTM D2166-98a (2000) [29] were carried out on samples treated with various additive contents. The curing times were 1, 7, 14, 35, and 90 days at optimum moisture content, the unit compaction energy was the same as that in a standard proctor test, and the length and diameter of each sample were 100 mm and 50 mm, respectively.

## 3. Results and Discussions

**Figure 2:**
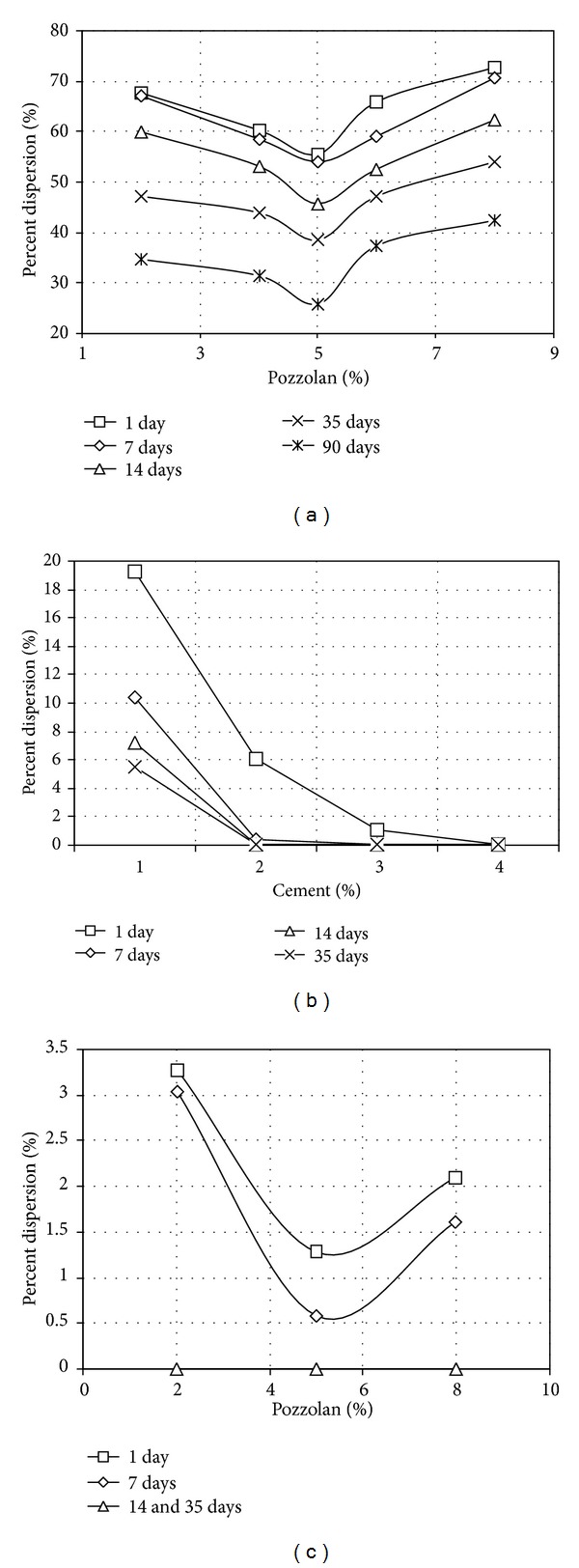
Percent Dispersion of samples treated; (a) with pozzolan contents and different curing times, (b) with cement contents and different curing times, and (c) with different pozzolan contents plus 1.5% cement content and different curing times.

The results of double hydrometer tests carried out on samples stabilized solely with pozzolan or Portland cement are given in Figures 2(a) and 2(b); while the results of double hydrometer tests carried out on samples stabilized simultaneously with various percentages of pozzolan and 1.5 percent Portland cement are given in Figure 2(c).

As given in Figure 2(a), the optimum pozzolan content for maximum reduction in dispersion potential of sample was 5 percent. The resulting dispersion potential from using 5 percent pozzolan, however, kept coming down with increasing curing time. Stabilization using pozzolan alone was apparently time consuming that even after 90 days, the dispersion potential kept decreasing with any given additional day of curing. Percent dispersion for a sample treated with 5 percent pozzolan and 90 days curing time was 26, which put the clay in the acceptable, non-dispersive category on the scale by Sherard and Decker (1977). Nevertheless, the mix could not achieve the fully non-dispersive status at the end of treatment process, even after 90 days. 

However, as indicated in Figure 2(b), when used alone, 1 to 4 percent Portland cement content was capable of reducing dispersion potential of samples down to zero percent. In the case of using pozzolan, the dispersion potential did not seem to have converged to zero even with any given amount of the additive used, but by using cement, the fully non-dispersive status was achieved. With 4 percent cement, the zero percent dispersion potential was attained after one day of curing; with 3 percent cement, the status was achieved in 7 days; and with 2 percent cement, it was achieved in 14 days. With 1 percent cement, the zero dispersion level could not be achieved regardless of the amount of curing time allowed; nevertheless, the sample was improved significantly into one of the non-dispersive categories. Thus 1.5 percent cement content was considered proper if it was to be used together and simultaneously with pozzolan, which is the cheaper treatment additive.

As indicated in Figure 2(c), the optimum pozzolan content in reducing dispersion potential was still 5 percent, even when used simultaneously with 1.5 percent cement. Moreover, the dispersion potential could now be reduced further in comparison to the case when pozzolan was used alone. Curing for 14 or 35 days has caused samples with simultaneous use of pozzolan and cement to achieve new levels of improvement with dispersion potential decreasing to almost zero percent. 

The qualitative categorization of dispersive clays after treatment with various additives and curing times is given in Table 2. The categorization criteria were based on standards which have been presented by Sherard and Decker (1977).

In the pinhole tests, the results were recorded in terms of cloudiness of flow, final flow rate, and erosion rate of the hole created in samples, with given hydraulic head. The summary of results and, categorization of treated specimens are given in Table 3. 

The use of 5 percent pozzolan with 90 days curing time has changed the designation of sample from D_1_ to ND_2_. The use of 3 percent cement with 7 days curing time has changed the designation of sample from D_1_ to ND_2_, and with 14 days curing the designation has changed to ND_1_. The use of 4 percent cement with 1 day curing time has changed the designation from D_1_ to ND_2_, and with 7 days curing time the designation has change to ND_1_. The use of 5 percent pozzolan and 1.5 percent cement with 1 day curing time has changed the designation from D_1_ to ND_2_, and with 14 days curing time the designation has change to ND_1_. The use of 2 percent pozzolan and 1.5 percent cement with 14 days curing time has changed the designation from D_1_ to ND_2_, and with 35 days of curing the designation has changed to ND_1_. 

**Figure 3:**
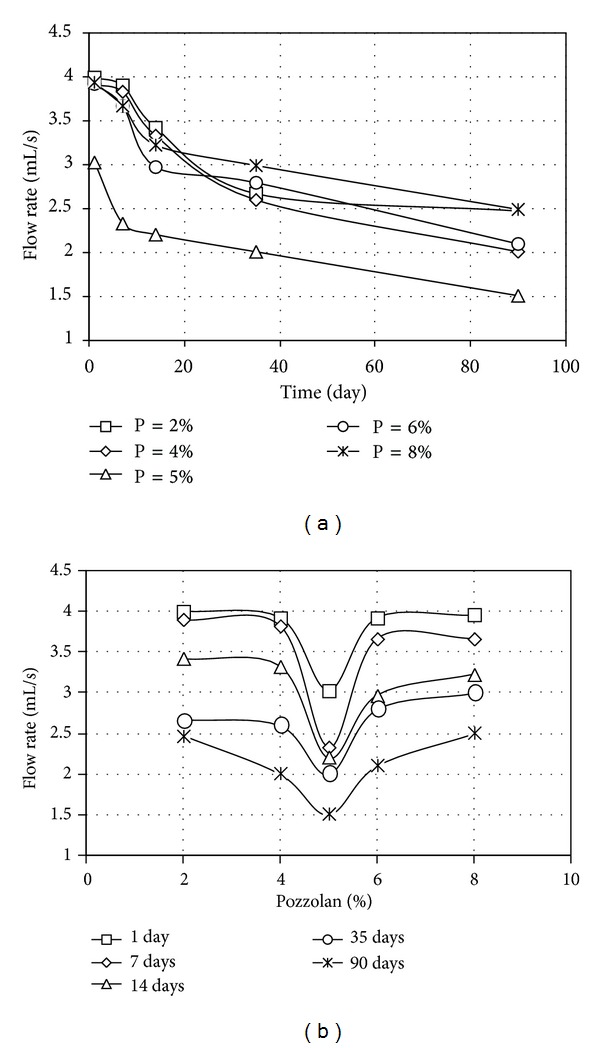
Flow rate for treated samples (a) of different pozzolan contents and (b) of different curing times, for different pozzolan contents.

**Figure 4:**
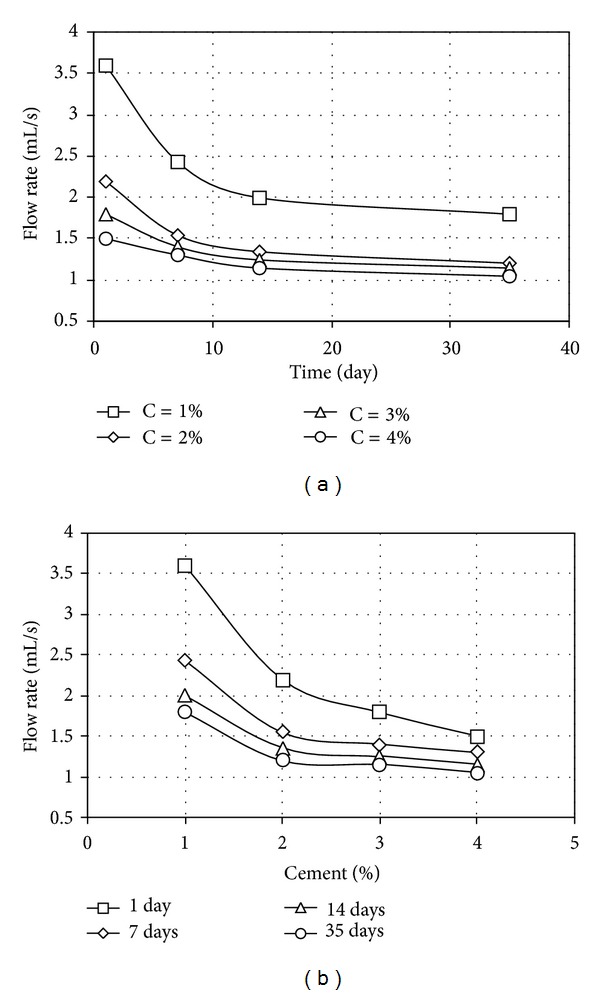
Flow rate for treated samples (a) of different cement contents and (b) of different curing times, for different cement contents.

**Figure 5:**
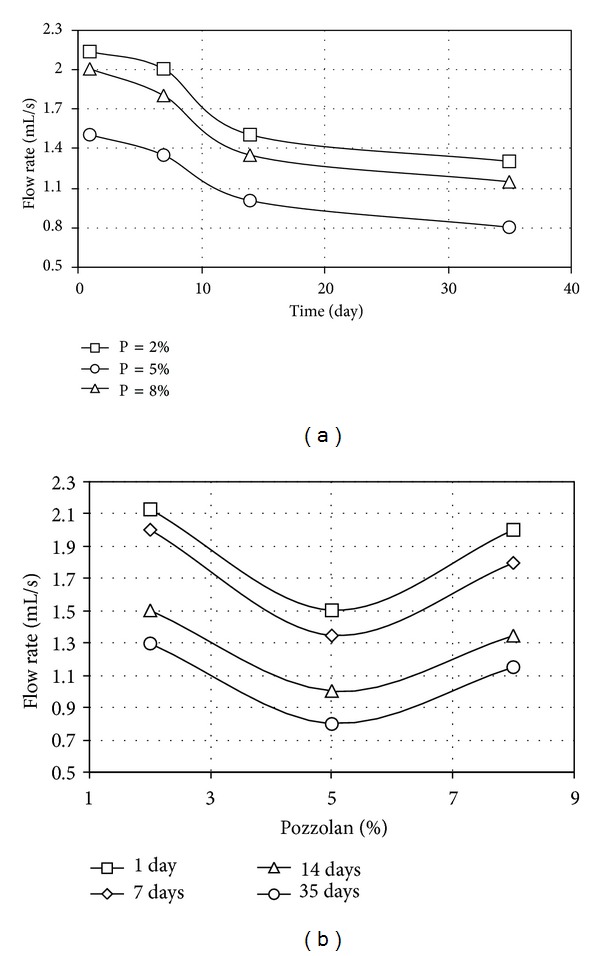
Flow rate for treated samples; (a) of different pozzolan contents plus 1.5% cement content, (b) of different curing times, for different pozzolan contents plus 1.5% cement content.

The results of pinhole tests carried out on samples stabilized individually with pozzolan and cement, in terms of final flow rates, are given in Figures 3(a), 3(b), 4(a), and 4(b) while the results of tests involving simultaneous use of various pozzolan percentages and 1.5 percent cement with various curing times are given, respectively, in Figures 5(a) and 5(b). 

As indicated in Figures 3(a) and 3(b), the optimum pozzolan contents corresponding to the lowest flow rates were 5 percent. The flow rates, however, decreased further with increasing curing time. 

Treatment with pozzolan only continued up to 90 days after application, and still the flow rate was decreased. Thus treatment with pozzolan alone could reduce dispersion potential down to an accepted level, although the sample did not become completely non-dispersive. Again, stabilization using pozzolan was a time consuming process.

As shown in Figures 4(a) and 4(b), the flow rates through the samples converged with singular cement content increasing from 1 to 4 percent. However, an increase from 2 to 4 percent in cement contents had caused only a little change in the flow rate, which led to the amount of 1.5 percent cement as optimum for use in the ensuing tests involving simultaneous application with pozzolan. 

As given in Figure 5(a), the flow rates through samples were lowest when 5 percent pozzolan content was used in addition to the 1.5 percent cement. Although the 5 percent optimum pozzolan content was the same as in the case of Figure 3(a) using pozzolan alone without the cement—the flow rate has been reduced further in comparison to the earlier tests. Another observation—the curves of Figure 5(a) appear to have similar shape although shifted into various positions with varying pozzolan contents. 

Figure 5(b) is a representation of Figure 5(a). The similarly shaped curves were once again evident although, respectively, shifted into various positions for different curing times—1, 7, 14, and 35 days—instead of different pozzolan contents. At 1.5 percent cement content, the optimum pozzolan content giving the lowest flow rate, for any curing time, was 5 percent, as noted before. The most significant reduction in flow rate has occurred for the interval between 7 and 14 days after improvement.

The classifications of various mixes based on the results of chemical tests were in accordance with criteria by Sherard et al. (1976). The resulting classifications are given in Table 4.

**Figure 6:**
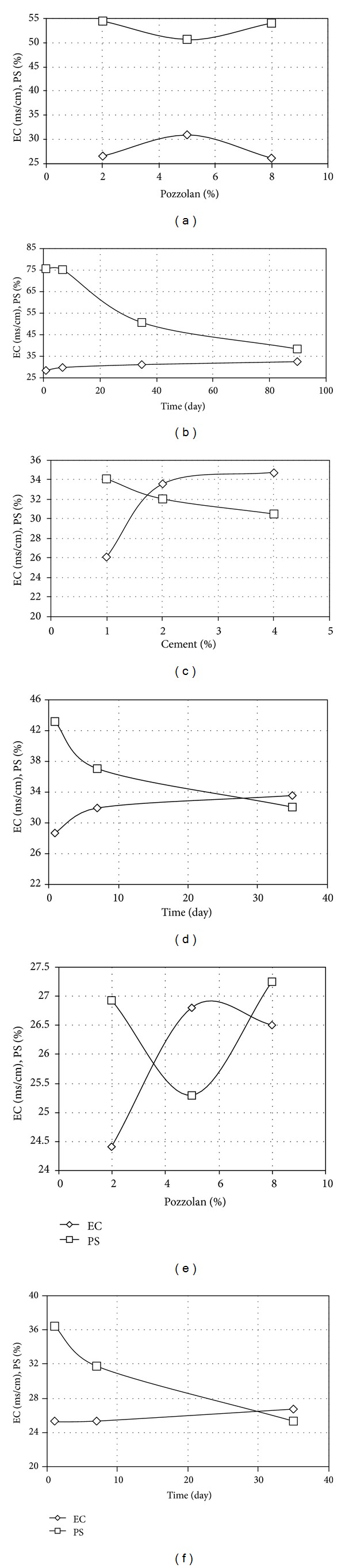
Electrical conductivity (EC) and percent sodium (PS) of samples treated: (a) with pozzolan and of 35 days curing time, (b) with 5% pozzolan and of various curing times, (c) with cement of 35 days curing time, (d) with 2% cement and of various curing times, (e) with 1.5% cement and various pozzolan percentages and of 35 days curing, and (f) with 1.5% cement and 5% pozzolan and of various curing times.

The variations of electrical conductivity (EC) and percent sodium (PS) of samples treated by pozzolan and cement, individually and simultaneously, are shown in Figures 6(a) to 6(f). Among samples treated by pozzolan alone, the one with 5 percent content has both the highest EC and the least PS, as given in Figure 6(a), which verifies again that the treatment led to the least dispersion potential. 

The ion exchange reaction apparently has reduced the double layer thickness within the clay structure. In an ion exchange reaction, pozzolan cations such as Ca^+2^ and Al^+3^ replace sodium cation, Na^+^, which is the specific feature of dispersive soils. Due to the replacement, the dispersive soil fabric changes to flocculated fabric, with decreased inter-particle repulsion and thus decreased dispersion potential.  Figure 6(b) shows the effect of curing time as a factor in the stabilization process. As curing time increased, EC increased and PS decreased, which suggests that with increasing curing time, ion exchange reaction progressed. The process appears to flatten as curing time approached 90 days.

Increasing cement content from 1 percent to 2 percent could significantly increase EC and therefore reduce dispersion potential as illustrated in Figure 6(c). Curing time factor has affected the stabilization process through adding cement alone, as presented in Figure 6(d). EC and PS appear to have changed rapidly during the first 7 days of curing time, although changes were still continuing after that. Modification with simultaneous addition of 5 percent pozzolan and 1.5 percent cement has produced a mix with lowest rate of PS recorded, as indicated in Figure 6(e). The treatment with simultaneous 5 percent pozzolan and 1.5 percent cement composition went on up until 35 days after curing, as shown in Figure 6(f). In other words, the change in the characteristics of samples treated with pozzolan and cement was a function of curing time as much as it was a function of pozzolan and cement percentages. Treatment by pozzolan alone required 90 days, by cement alone 7 to 14 days, and by simultaneous composition of pozzolan and cement 14 to 35 days. 

**Figure 7:**
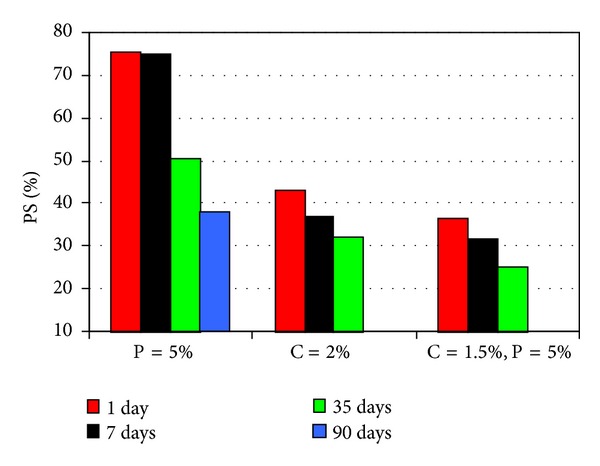
Comparison of results from chemical tests.

**Figure 8:**
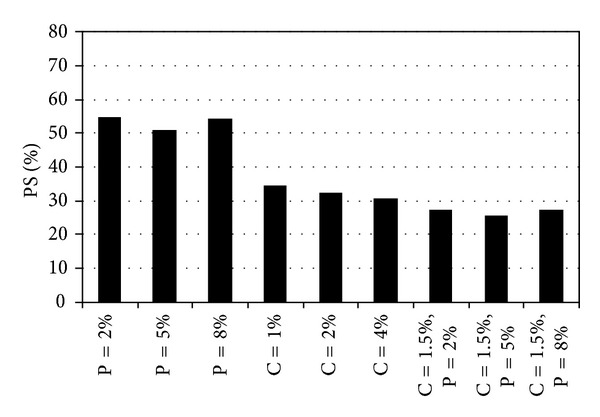
Comparison of results from chemical tests of 35 days curing time.

**Figure 9:**
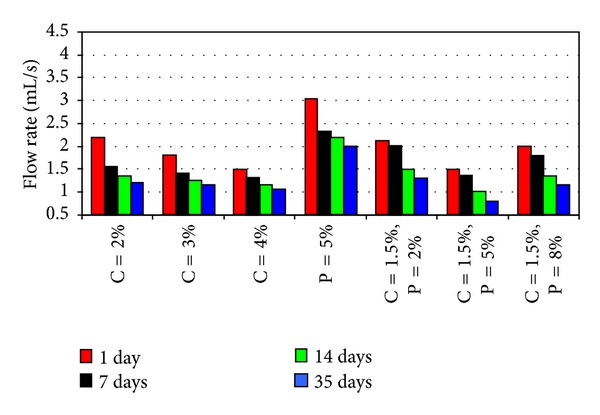
Comparison of results from pinhole tests.

The results from pinhole and chemical tests were compared in Figures 7, 8, and 9 and the following features were noted.

1. At the end of the first day, among all treated samples, those mixed with 4 percent cement alone, and with 1.5 percent cement plus 5 percent pozzolan simultaneously have given the lowest dispersion potentials, which demonstrates the quick effect brought about by the cement in the treatment.
2. The best overall result in reducing dispersion potential has come from one with 1.5 percent cement plus 5 percent pozzolan and of 35 days of curing. A mixture with such compositions was better than any one singly treated with cement or pozzolan.
3. In general, if used alone in controlling dispersive soils, cement was better than pozzolan. Treatment with 2 percent cement and 7 days of curing has produced a completely nondispersive soil. On the other hand, treatment with 5 percent pozzolan has taken 90 days of curing in producing a non-dispersive soil.

**Figure 10:**
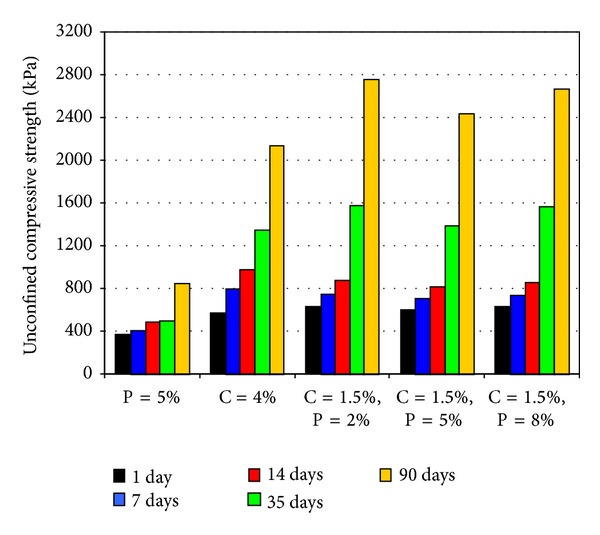
Comparison of results from unconfined compressive strength tests.

A final verification that clay has been optimally improved by treatment with simultaneous 1.5 percent cement plus 5 percent pozzolan can be seen from the results of unconfined compressive tests carried out on samples treated with various pozzolan and cement contents, as given in Figure 10. Nevertheless, in this case, the optimum simultaneous pozzolan content corresponding to maximum unconfined compressive strength (UCS) was 2 percent, to be more exact, and not 5 percent. 

Apparently, clay treated for best erosion resistance was not necessarily the best in terms of physical strength. Note also that after 90 days, treatments with 5 percent pozzolan a lone and 4 percent cement a lone, respectively, have increased the strengths to 2.6 and 6.7 times of the original untreated capacities. On the other hand, for the same curing period, treatments with 5 percent pozzolan plus 1.5 percent cement and 2 percent pozzolan plus 1.5 percent cement have multiplied the strengths by 7.6- and 8.6- folds, respectively. Simultaneous use of pozzolan and cement has increased physical strength of clay as it has improved resistance against erosion.

## 4. Conclusions

The best treatment procedure must be economically sound too. Due to the higher cost of cement as compared to that of pozzolan, replacing some cement for pozzolan has appeared to have merits. The study has shown that meagre 1.5 percent cement along with 5 percent pozzolan would provide beneficial results in the treatment of dispersive clays. 

In this study, with given optimum amounts of cement and pozzolan added simultaneously to dispersive clays, the ion exchanges that have taken place between Ca^2+^ and Al^3+^ of the additives and the Na^+^ of the clay have flocculated the fabrics, decreased dispersion problem, increased EC, decreased PS, and brought about general improvement to the soil. However, treated clay with the best resistance against dispersion or erosion was not necessarily the best against physical stress, to be exact. 

## Figures and Tables

**Table 1:** Properties of clay from Azerbaijan which has been altered to become fully dispersive.

	Standard	Value
Geotechnical properties		
Sand (%)	ASTM D 422-63 [24]	2
Silt fraction (%)	ASTM D 422-63 [24]	36
Clay fraction (%)	ASTM D 422-63 [24]	62
Liquid limit (%)	ASTM D 4318-98 [25]	34
Plastic limit (%)	ASTM D 4318-98 [25]	17
Plasticity index (%)	ASTM D 4318-98 [25]	17
Soil classification	ASTM D 2487-98 [26]	CL
Specific gravity (Gs)	ASTM D 854-98 [27]	2.79
Optimum moisture content (%)	ASTM D 698-91 [28]	16.5
Maximum dry density (g/cm^3^)	ASTM D 698-91 [28]	1.82
Unconfined compressive strength (kPa)	ASTM D 2166-98a [29]	320
Chemical properties		
PH	ASTM D 2967-71 [30]	8.5
Na^+^, (meq/lit)	ASTM D 4542-95 [15]	179.16
K^+^, (meq/lit)	ASTM D 4542-95 [15]	16.89
Ca^2+^, (meq/lit)	ASTM D 4542-95 [15]	21.25
Mg^2+^, (meq/lit)	ASTM D 4542-95 [15]	13.15
EC (Electrical conductivity), (ms/cm)	—	20.9
TDS (Total dissolved salts: Na^+^ + Ca^2+^ + Mg^2+^ + K^+^), (meq/lit)	—	230.45
SAR (Sodium absorption ratio), (meq/lit)	—	43.2
PS (Percent sodium over total dissolved salts)	—	78
Dispersivity properties		
Percent dispersion by double hydrometer test	ASTM D 4221-99 [13]	100
Call name		Fully dispersive clay
Classification by double hydrometer test	ASTM D 4221-99 [13]	Dispersive
Classification by pinhole test	ASTM D 4647-93 [14]	Dispersive (D_1_)
Classification by chemical test	Sherard et al., 1976 [16]	Dispersive

**Table 2:** Categorization based on double hydrometer test results of clay sample stabilized with pozzolan, cement, and pozzolan plus 1.5 percent cement.

Additive	Curing time				
	1 day	7 days	14 days	35 days	90 days
Pozzolan 2%	Dispersive	Dispersive	Dispersive	Intermediate	Intermediate
Pozzolan 4%	Dispersive	Dispersive	Dispersive	Intermediate	Intermediate
Pozzolan 5%	Dispersive	Dispersive	Intermediate	Intermediate	Nondispersive
Pozzolan 6%	Dispersive	Dispersive	Dispersive	Intermediate	Intermediate
Pozzolan 8%	Dispersive	Dispersive	Dispersive	Dispersive	Intermediate
Cement 1%	Nondispersive	Nondispersive	Nondispersive	Nondispersive	Nondispersive
Cement 2%	Nondispersive	Nondispersive	Nondispersive	Nondispersive	Nondispersive
Cement 3%	Nondispersive	Nondispersive	Nondispersive	Nondispersive	Nondispersive
Cement 4%	Nondispersive	Nondispersive	Nondispersive	Nondispersive	Nondispersive
Pozzolan 2% + 1.5% cement	Nondispersive	Nondispersive	Nondispersive	Nondispersive	Nondispersive
Pozzolan 5% + 1.5% cement	Nondispersive	Nondispersive	Nondispersive	Nondispersive	Nondispersive
Pozzolan 8% + 1.5% cement	Nondispersive	Nondispersive	Nondispersive	Nondispersive	Nondispersive

**Table 3:** Categorization based on pinhole test results of clay sample stabilized with pozzolan alone, cement alone, and pozzolan plus 1.5 percent cement.

Additive	Curing time				
	1 day	7 days	14 days	35 days	90 days
Pozzolan 2%	D_2_	D_2_	ND_4_	ND_3_	ND_3_
Pozzolan 4%	ND_4_	ND_4_	ND_4_	ND_3_	ND_3_
Pozzolan 5%	ND_3_	ND_3_	ND_3_	ND_3_	ND_2_
Pozzolan 6%	ND_4_	ND_4_	ND_3_	ND_3_	ND_3_
Pozzolan 8%	D_2_	ND_4_	ND_3_	ND_3_	ND_3_
Cement 1%	ND_4_	ND_3_	ND_3_	ND_3_	ND_3_
Cement 2%	ND_3_	ND_2_	ND_2_	ND_2_	ND_2_
Cement 3%	ND_3_	ND_2_	ND_1_	ND_1_	ND_1_
Cement 4%	ND_2_	ND_1_	ND_1_	ND_1_	ND_1_
Pozzolan 2% + 1.5% cement	ND_3_	ND_3_	ND_2_	ND_1_	ND_1_
Pozzolan 5% + 1.5% cement	ND_2_	ND_2_	ND_1_	ND_1_	ND_1_
Pozzolan 8% + 1.5% cement	ND_3_	ND_3_	ND_2_	ND_1_	ND_1_

Note D_1_ and D_2_: dispersive; ND_1_ and ND_2_: nondispersive; ND_3_ and ND_4_: intermediate.

**Table 4:** Categorization based on chemical test results of clay sample stabilized with pozzolan, cement, and pozzolan plus 1.5 percent cement.

Additive	Curing time				
	1 day	7 days	14 days	35 days	90 days
Pozzolan 2%				Intermediate	Intermediate
Pozzolan 5%	Dispersive	Dispersive		Intermediate	Nondispersive
Pozzolan 8%				Intermediate	Intermediate
Cement 1%				Nondispersive	Nondispersive
Cement 2%	Intermediate	Intermediate	Nondispersive	Nondispersive	Nondispersive
Cement 3%				Nondispersive	Nondispersive
Cement 4%				Nondispersive	Nondispersive
Pozzolan 2% + 1.5% cement				Nondispersive	Nondispersive
Pozzolan 5% + 1.5% cement	Nondispersive	Nondispersive	Nondispersive	Nondispersive	Nondispersive
Pozzolan 8% + 1.5% cement				Nondispersive	Nondispersive
